# Lower hepatocellular carcinoma surveillance in metabolic dysfunction‐associated steatotic liver disease: Impact on treatment eligibility

**DOI:** 10.1111/jgh.16727

**Published:** 2024-08-27

**Authors:** Connor Henry‐Blake, Vinay Balachandrakumar, Mohamed Kassab, Joshua Devonport, Charmaine Matthews, James Fox, Elisabeth Baggus, Alexander Henney, Nicholas Stern, Daniel J Cuthbertson, Daniel Palmer, Philip J Johnson, David M Hughes, Theresa J Hydes, Timothy J S Cross

**Affiliations:** ^1^ Department of Gastroenterology and Hepatology Liverpool University Hospitals NHS Foundation Trust Liverpool UK; ^2^ Department of Diabetes and Endocrinology Liverpool University Hospitals NHS Foundation Trust Liverpool UK; ^3^ Department of Cardiovascular and Metabolic Medicine University of Liverpool Liverpool UK; ^4^ Liverpool Centre for Cardiovascular Sciences University of Liverpool and Liverpool University Hospitals NHS Foundation Trust Liverpool UK; ^5^ Department of Molecular and Clinical Cancer Medicine University of Liverpool Liverpool UK; ^6^ Department of Health Data Science, Institute of Population Health University of Liverpool Liverpool UK

**Keywords:** early detection of cancer, hepatocellular carcinoma, non‐alcoholic fatty liver disease

## Abstract

**Background and Aim:**

This study aimed to compare the determinants and impact of hepatocellular carcinoma (HCC) surveillance rates for people with metabolic dysfunction‐associated steatotic liver disease (MASLD) *versus* other chronic liver diseases.

**Methods:**

A dataset of HCC patients from a UK hospital (2007–2022) was analyzed. The Mann–Whitney *U*‐test compared continuous variables. The *χ* ^2^ and two‐tailed Fisher exact tests compared categorical data. Regression modeling analyzed the impact of MASLD on the size and number of HCC nodules and curative treatment. The Cox proportional hazards model assessed the influence of MASLD on overall survival.

**Results:**

A total of 176 of 687 (25.6%) HCC patients had MASLD. Fewer people with MASLD HCC were enrolled in HCC surveillance compared to non‐MASLD HCC (38 [21.6%] *vs* 215 [42.1%], *P* < 0.001). Patients with MASLD HCC were less likely to have been under secondary care (*n* = 57 [32.4%] *vs* 259 [50.7%], *P* < 0.001) and less likely to have cirrhosis (*n* = 113 [64.2%] *vs* 417 [81.6%], *P* < 0.001). MASLD was associated with a 12.3‐mm (95% confidence interval [CI] 10.8–14.0 mm) greater tumor diameter compared to people without MASLD (*P* = 0.002). Patients with MASLD HCC had 0.62 reduced odds (95% CI 0.43–0.91) of receiving curative treatment compared to non‐MASLD HCC (*P* = 0.014). Overall survival was similar for patients with MASLD HCC *versus* non‐MASLD HCC (hazard ratio 1.03, 95% CI 0.85–1.25, *P* = 0.748).

**Conclusion:**

Patients with MASLD are less likely to have been enrolled in HCC surveillance due to undiagnosed cirrhosis or presenting with non‐cirrhotic HCC. Patients with MASLD HCC present with larger tumors and are less likely to receive curative treatment.

## Graphical abstract

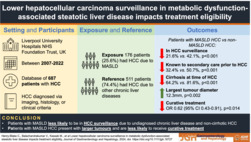

## Introduction

The incidence and mortality rates of hepatocellular carcinoma (HCC) have risen in stark contrast to other cancers.^1^ Furthermore, it is predicted that HCC incidence rates will increase by 55% between 2020 and 2040.^2^ Metabolic dysfunction‐associated steatotic liver disease (MASLD) is driven by the obesity and type 2 diabetes epidemics. MASLD has been identified as the fastest‐growing cause of HCC in liver transplant candidates.^3^ Estes *et al*. forecast that the incidence of MASLD‐related HCC will increase by 137% by 2030 in the USA.^4^ A recent meta‐analysis has identified that in people with MASLD cirrhosis, the incidence rate of HCC is 3.79 per 100 person years.^5^ Incidence rates are significantly lower for people with MASLD without cirrhosis (0.3 per 100 person years).^6^ Overall survival rates for HCC are low,^7^ yet curative treatment is possible with early detection.^7^, ^8^ One‐year survival rates for HCC are 78% *versus* 20% for those diagnosed at the earliest *versus* latest stage.^9^ Although clinical trial evidence for HCC surveillance in Western populations is lacking, meta‐analyses suggest that surveillance can lead to earlier cancer detection and improved survival in people with cirrhosis.^10^

A lower proportion of patients with MASLD‐related HCC undergo surveillance compared to those who develop HCC secondary to other chronic liver diseases. A meta‐analysis of 61 studies (94 636 patients) identified that only 32.8% of people with MASLD HCC had undergone HCC surveillance in comparison to 55.7% of people with HCC due to other causes (*P* < 0.0001).^11^ While there were no significant differences in treatment allocation or overall survival, patients with MASLD HCC experienced reduced disease‐free survival. However, significant heterogeneity was reported between the studies. Furthermore, studies did not explore reasons for lower rates of HCC surveillance in MASLD, making it vital for health‐care providers to understand and address this gap in care.

We aimed to compare HCC surveillance rates for different etiologies of chronic liver disease using a large, well‐phenotyped UK cohort, exploring barriers to HCC surveillance and variations in rates of early HCC detection, treatment, and overall survival.

## Methods

### Data collection

A dataset of adults with HCC diagnosed at Liverpool University Hospitals NHS Foundation Trust (LUHFT) was developed (February 2007–April 2022). LUHFT, situated in Northwest England, comprises two large teaching hospitals (Royal Liverpool University Hospital and Aintree University Hospital). Data were collected prospectively via attendances at a specialist hepatobiliary cancer clinic (Royal Liverpool University Hospital) and retrospectively (Aintree University Hospital) from hepatobiliary cancer multidisciplinary meeting records. This dataset describes patients with an established diagnosis of HCC presenting via different routes, including the HCC surveillance program. The Trust's surveillance policy is guideline led.^12^, ^13^ Patients with cirrhosis (all etiologies) or hepatitis B viral infection at high risk of HCC who are suitable for treatment are offered 6 months of ultrasound surveillance.

Data were collected on demographics, etiology, and stage of chronic liver disease, HCC characteristics (largest lesion diameter, number of lesions, vascular invasion, distant metastasis, and alpha‐fetoprotein at diagnosis), the Barcelona Clinic Liver Cancer (BCLC) stage, Eastern Cooperative Oncology Group performance status, primary treatment planned/received, and date of death. The model for end‐stage liver disease‐Na score and albumin–bilirubin grade were calculated from the time of HCC diagnosis. For both sites, data were collected retrospectively on metabolic disease, comorbidities, liver disease stage at the time of HCC diagnosis, and prior contact with secondary care hepatology services.

### Definitions

The MASLD diagnosis was based on radiological evidence of steatosis or cryptogenic cirrhosis, with at least one metabolic risk factor and without a history of significant alcohol intake or another cause of chronic liver disease.^14^ Other causes of chronic liver disease (alcohol‐related liver disease [ALD], chronic viral hepatitis, autoimmune, and inherited liver disease) were identified according to standard definitions used in clinical practice. For people with cirrhosis where no cause could be found or with insufficient data on etiology, diagnoses of cryptogenic cirrhosis or cirrhosis of unknown cause were made, respectively.

A diagnosis of cirrhosis was made based on imaging, histology, or clinical criteria. HCC was diagnosed by radiologists using internationally agreed criteria for contrast‐enhanced cross‐sectional imaging, that is, magnetic resonance imaging or computed tomography. Where the patient did not have cirrhosis diagnosed or the imaging was non‐diagnostic, a targeted liver biopsy was performed.^12^ All patients with HCC were discussed within a multidisciplinary team. Patients were recorded as known to secondary care services if they had attended a hepatology appointment in the preceding 12 months before HCC diagnosis or were under HCC surveillance.

### Data analysis

Demographic variables were not normally distributed (Kolmogorov–Smirnov test). Continuous variables were described using median and interquartile ranges (IQRs), and differences between MASLD and non‐MASLD groups were reported using the Mann–Whitney *U*‐test. Categorical data were compared using the *χ* ^2^ test or the two‐tailed Fisher exact test, where expected counts were < 5. Missing data were excluded using pairwise exclusion.

Key covariates were selected based on the literature as independent variables for the regression analyses and constructed into three models: model 1 (age and sex), model 2 (age, sex, and diabetes status), and model 3 (age, sex, diabetes status, and HCC surveillance status). Deprivation status was not included, as in this population, deprivation was not significantly associated with HCC size, nodule number, or overall survival and contained > 10% missing data. Missing data were excluded listwise to ensure comparability between models.

The impact of HCC etiology (MASLD *vs* non‐MASLD) on the log diameter of the largest tumor (continuous scale) was assessed via linear regression. The log diameter was used as the residuals of HCC diameter were not normally distributed. Coefficients and 95% confidence intervals (CIs) are presented. The coefficients of the log diameter were exponentiated to determine the impact of each clinical variable on the change in HCC diameter in millimeters.

Logistic regression and negative binomial regression assessed the impact of HCC etiology on having a tumor diameter > 5 cm and the overall number of tumors, respectively. The impact of an MASLD diagnosis on the likelihood of receiving curative treatment was assessed via logistic regression. The Cox proportional hazards model assessed the influence of a diagnosis of MASLD on overall survival from the point of HCC diagnosis. Kaplan–Meier curves compared survival rates. A *P* value of < 0.05 determined statistical significance. Data analysis was performed using IBM SPSS software v.29.0.

### Ethics

This study was registered with the LUHFT NHS Trust Clinical Audit Management Department (audit number 11630).

## Results

### Study population

**Figure 1:**
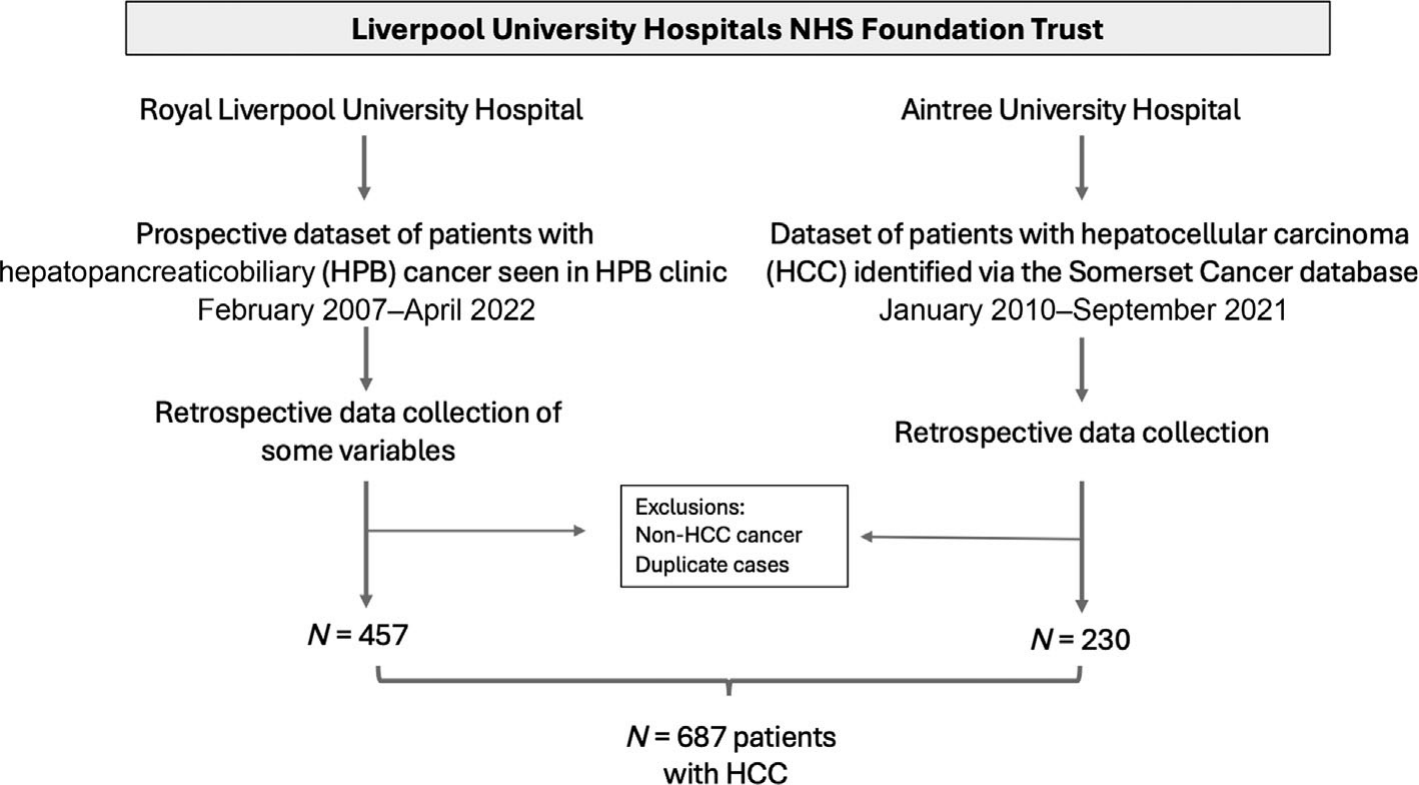
Study flow chart.

**Figure 2:**
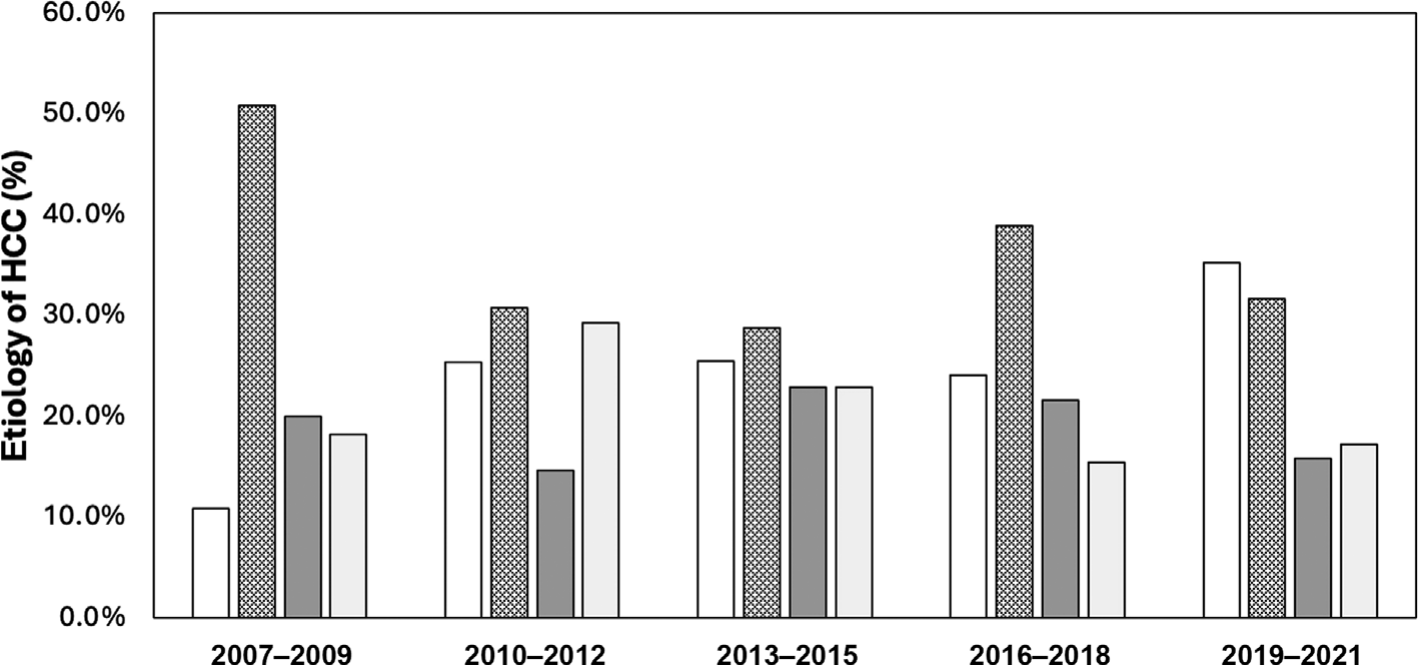
Temporal trends in the etiological drivers of hepatocellular carcinoma (HCC). 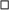, metabolic dysfunction‐associated steatotic liver disease; 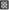, alcohol‐related liver disease; 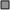, hepatitis C virus infection; 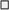, other.

In total, 687 patients were diagnosed with HCC (Fig. 1). The median age was 68 years (IQR 16), and 76.7% were men. Within this cohort, 176 (25.6%) had MASLD, 233 (33.9%) had ALD, 135 (19.8%) had a hepatitis C virus (HCV) infection, and 143 (20.8%) had another chronic liver disease. Within the non‐MASLD group, 43 (8.4%) did not have sufficient data to make a clear etiological diagnosis, and 21 (4.1%) had a diagnosis of “non‐alcoholic fatty liver disease” without a documented metabolic risk factor for MASLD. Patient characteristics are shown (Table 1). Patients presenting with MASLD HCC were older and had higher rates of obesity, type 2 diabetes, and cardiovascular disease compared to other etiologies. Liver function determined via albumin, bilirubin, prothrombin time, and platelet count was statistically better for people with MASLD HCC, and alpha‐fetoprotein levels at the time of presentation with HCC were comparable between groups. Over the data collection period, the frequency of MASLD‐related HCC cases consistently increased from 10.9% (2007–2009) to 35.3% (2019–2021) (Fig. 2).

**Table 1:** Baseline demographics at the time of hepatocellular carcinoma diagnosis according to the etiology of chronic liver disease

	Overall cohort	MASLD	Non‐MASLD	*P* value
*n* (%)	687	176 (25.6)	511 (74.4)	
Demographics				
Age (years), median (IQR)	68 (16)	74.0 (12)	68.0 (17)	< 0.001
Male, *n* (%)	527 (76.7)	134 (76.1)	393 (76.9)	0.835
White ethnicity, *n* (%)	639 (93.0)	165 (93.8)	474 (92.8)	0.641
*Ethnicity, missing data*	8 (1.2)	2 (1.1)	6 (1.2)	
Lowest multiple deprivation index decile, *n* (%)	281 (40.9)	61 (34.7)	220 (43.1)	0.128
*Deprivation, missing data*	69 (10.0)	24 (13.6)	45 (8.8)	
Metabolic disease				
Overweight/obesity, *n* (%)	275 (40.0)	111 (63.1)	164 (32.1)	< 0.001
*Overweight status, missing data*	259 (37.7)	45 (25.6)	214 (41.9)	
BMI, median (IQR)	27.5 (7.3)	29.9 (7.9)	27.4 (7.0)	< 0.001
< 25 (%)	113 (16.4)	15 (8.5)	98 (19.2)	< 0.001
25–30 (%)	131 (19.0)	49 (27.8%)	82 (16.0)	< 0.040
> 30 (%)	113 (16.4)	46 (26.17)	67 (13.1)	< 0.006
*BMI, missing data*	330 (48.0)	66 (37.5)	264 (51.7)	
Type 2 diabetes, *n* (%)	279 (40.6)	126 (71.6)	153 (29.9)	< 0.001
*Type 2 diabetes, missing data*	14 (2.0)	0 (0.0)	14 (2.7)	
HbA1c (mmol/mol), median (IQR) in people with type 2 diabetes	47 (25.0)	44.0 (27.0)	44.0 (27.0)	< 0.001
*HbA1c, missing data*	342 (49.8)	45 (25.6)	297 (58.1)	
Comorbidities, *n* (%)				
Cardiovascular disease	148 (21.5)	64 (36.4)	84 (16.4)	< 0.001
Respiratory disease	91 (13.2)	23 (13.1)	68 (13.3)	0.936
Extrahepatic cancer	50 (7.3)	13 (7.4)	37 (7.2)	0.949
Chronic kidney disease	32 (4.7)	12 (6.8)	20 (3.9)	0.115
Cirrhosis	530 (77.1)	113 (64.2)	417 (81.6)	< 0.001
Pathology results				
Bilirubin, μmol, median (IQR)	16 (19)	11.0 (11.0)	15 (16)	< 0.001
*Bilirubin count, missing data*	7 (1.0)	0 (0.0)	7 (1.4)	
Albumin, mg/L, median (IQR)	38 (8.0)	40.0 (8)	39.0 (8)	0.021
*Albumin count, missing data*	7 (1.0)	0 (0.0)	7 (1.4)	
Platelets (×10^9^/L), median (IQR)	155 (124)	199.0 (115)	132.5 (111.0)	< 0.001
*Platelet count, missing data*	9 (1.3)	1 (0.6)	8 (1.6)	
Prothrombin time (s), median (IQR)	12.3 (2.0)	12.0 (2)	12.6 (2)	0.002
*Prothrombin time, missing data*	31 (4.5)	9 (5.1)	22 (4.3)	
Alpha‐fetoprotein (IU/mL), median (IQR)	9 (180)	7.0 (90.0)	6.0 (32)	0.155
*Alpha‐fetoprotein, missing data*	48 (7.0)	11 (6.3)	37 (7.2)	
Alanine transaminase, μmol, median (IQR)	35 (31)	32 (28)	35 (33)	0.164
*Alanine transaminase, missing data*	203 (29.5)	43 (24.4)	160 (31.3)	
Aspartate transaminase, μmol, median (IQR)	44 (33)	42 (29)	45 (39)	0.389
*Aspartate transaminase, missing data*	416 (60.6)	97 (55.1)	319 (62.4)	
Fibrosis‐4 score, μmol, median (IQR)	3.8 (3.7)	3.4 (3.5)	4.0 (4.0)	0.226
*Fibrosis‐4 score, missing data*	418 (60.8)	97 (55.1)	321 (62.8)	

BMI, body mass index; HbA1c, glycated hemoglobin; IQR, interquartile range; MASLD, metabolic dysfunction‐associated steatotic liver disease.

### People with metabolic dysfunction‐associated steatotic liver disease are less likely to have hepatocellular carcinoma detected within a surveillance program

**Figure 3:**
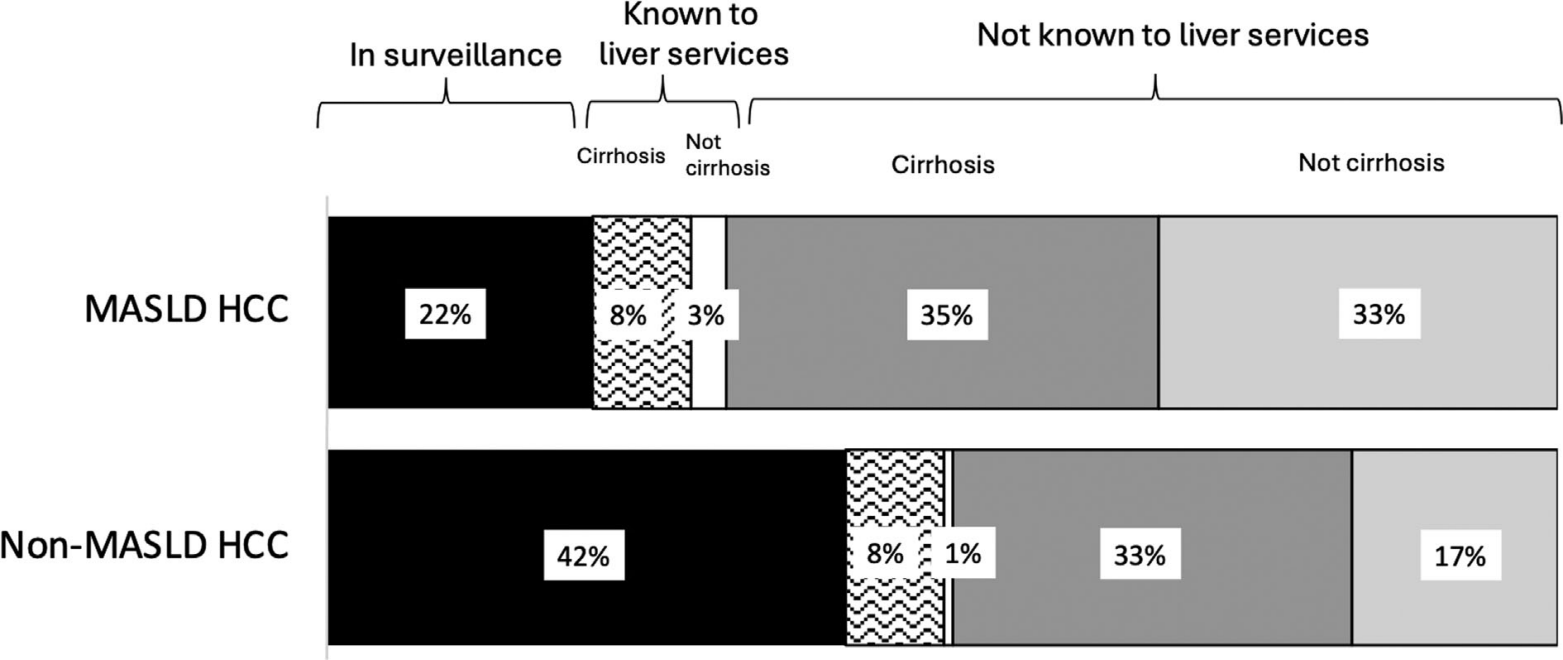
Frequency of people presenting with a hepatocellular carcinoma (HCC) who had previously been enrolled in surveillance and barriers towards HCC surveillance according to disease etiology. MASLD, metabolic dysfunction‐associated steatotic liver disease.

Only 38 (21.6%) people with MASLD HCC had been enrolled in HCC surveillance before cancer diagnosis, compared with 215 (42.1%) people with non‐MASLD‐related HCC (*P* < 0.001) (Table S1 and Fig. 3).

For the whole study cohort, 186 (27.5%) patients had their HCC detected via imaging (ultrasound, computed tomography, or magnetic resonance imaging) that was requested for surveillance. For people with MASLD, 26 (14.8%) of scans that detected HCC were performed for surveillance, versus 160 (32.0%) for non‐MASLD HCC (*P* < 0.001). Other diagnostic routes, including symptomatic presentations, abnormal biochemistry, and incidental radiological findings, are described (Table S2).

### Reasons for the low rates of hepatocellular carcinoma surveillance

For the whole cohort (patients presenting within and outside of a surveillance program), only 57 (32.4%) patients with MASLD were known to receive secondary care liver services before HCC diagnosis, compared to 259 (50.7%) people with another etiology of chronic liver disease (*P* < 0.001) (Table S1 and Fig. 3). Furthermore, only 113 (64.2%) patients with MASLD HCC had cirrhosis at diagnosis, compared to 417 (81.6%) patients with non‐MASLD HCC (*P* < 0.001). For people with MASLD who presented with HCC directly from primary care, 57 (47.9%) did not have pre‐existing cirrhosis at HCC presentation, compared to 85 (33.9%) for non‐MASLD HCC (*P* = 0.010).

### Hepatocellular carcinoma present with a more advanced Barcelona Clinic Liver Cancer stage and larger tumors compared to hepatocellular carcinoma related to other etiologies of chronic liver disease

Overall, 52 (29.5%) people with MASLD HCC presented with BCLC stage 0/A compared to 200 (39.1%) patients with HCC due to a non‐MASLD etiology (*P* = 0.025) (Table 2). Patients with MASLD were more likely to present at BCLC stage B (*n* = 59, 33.5%, *vs n* = 116, 22.7%, *P* = 0.004) or C (*n* = 30, 17.0%, *vs n* = 55, 10.8%, *P* = 0.028) than people with non‐MASLD HCC.

**Table 2:** Stage of HCC at the time of diagnosis according to disease etiology

	Overall cohort	MASLD	Non‐MASLD	*P* value
Number of HCC tumors				
< 3 tumors, *n* (%)	488 (71.0)	124 (70.5)	364 (71.4)	0.817
3–5 tumors, *n* (%)	111 (16.2)	27 (15.3)	84 (16.4)	0.726
> 5 tumors, *n* (%)	87 (12.7)	25 (14.2)	62 (12.1)	0.482
*Number of HCC tumors, missing data*	1 (0.1)	0 (0.0)	1 (0.2)	
Diameter largest tumor				
Median (mm) (IQR)	30 (34.0)	44 (45.0)	30 (40.0)	< 0.001
Tumor > 5 cm, *n* (%)	205 (29.8)	70 (39.8)	135 (26.4)	< 0.001
*Tumor diameter, missing data*	19 (2.8)	5 (2.8)	14 (2.7)	
Invasion				
Regional lymphadenopathy, *n* (%)	132 (19.2)	35 (19.9)	97 (19.0)	0.801
*Lymphadenopathy, missing data*	1 (0.1)	0 (0.0)	1 (0.2)	
Invasion of adjacent organs, *n* (%)	16 (2.3)	6 (3.4)	10 (2.0)	0.292
*Organ invasion, missing data*	12 (1.7)	0 (0.0)	12 (2.3)	
Vascular invasion, *n* (%)	116 (16.9)	24 (13.6)	92 (18.0)	0.147
*Vascular invasion, missing data*	12 (1.7)	0 (0.0)	12 (2.3)	
Metastases, *n* (%)	97 (14.1)	27 (15.3)	70 (13.7)	0.596
*Metastases, missing data*	1 (0.1)	0 (0.0)	1 (0.2)	
BCLC stage				
0, *n* (%)	36 (5.2)	5 (2.8)	31 (6.1)	0.099
A, *n* (%)	216 (31.4)	47 (26.7)	169 (33.1)	0.123
0/A, *n* (%)	252 (36.7)	52 (29.5)	200 (39.1)	0.025
B, *n* (%)	175 (25.5)	59 (33.5)	116 (22.7)	0.004
C, *n* (%)	85 (12.4)	30 (17.0)	55 (10.8)	0.028
D, *n* (%)	173 (25.2)	34 (19.3)	139 (27.2)	0.040
*BCLC stage, missing data*	2 (0.3)	1 (0.6)	1 (0.2)	

BCLC stage, Barcelona Clinic Liver Cancer stage; HCC, hepatocellular carcinoma; IQR, interquartile range; MASLD, metabolic dysfunction‐associated steatotic liver disease.

At the time of HCC diagnosis, the median diameter of the largest tumor for people with HCC due to MASLD was 44 mm (IQR 45 mm), compared to 30 mm (IQR 40 mm) (*P* < 0.001) for people with HCC secondary to non‐MASLD etiologies (Table 2). Overall, 70 (39.8%) individuals with MASLD HCC had the largest tumor diameter > 5 cm in comparison to 135 (26.4%) people with non‐MASLD HCC (*P* < 0.001).

Linear regression identified that a diagnosis of MASLD was associated with a 12.3‐mm (95% CI 10.8–14.0 mm) greater tumor diameter compared to people without MASLD (*P* = 0.002) (Table S3). This association remained statistically significant following adjustment for age, sex, and type 2 diabetes, but not surveillance status. Similarly, in logistic regression modeling, MASLD etiology was associated with a greatest HCC tumor diameter of > 5 cm (odds ratio 1.88, 95% CI 1.31–2.71, *P* < 0.001) (Table S4). This association remained statistically significant following adjustment for age, sex, and diabetes status (odds ratio 1.84, 95% CI 1.214–2.774, *P* < 0.004) and was lost after adjustment for enrollment in HCC surveillance. Indeed, entry into HCC surveillance was associated with a 28% decreased risk of presenting with the largest tumor diameter of > 5 cm (*P* < 0.001). A diagnosis of MASLD did not influence the overall number of tumors in unadjusted or adjusted models (Table S5).

### Patients with metabolic dysfunction‐associated steatotic liver disease hepatocellular carcinoma have better liver function and similar performance status compared to patients with non‐metabolic dysfunction‐associated steatotic liver disease hepatocellular carcinoma

In total, 113 (64.2%) patients with MASLD presented with cirrhosis at the time of HCC diagnosis, compared to 417 (81.6%) for non‐MASLD chronic liver disease (*P* < 0.001) (Table 3). For those with cirrhosis, frequencies of a model for end‐stage liver disease score < 9 were similar between groups (*n* = 54, 47.8%, for MASLD HCC and *n* = 119, 47.7%, for non‐MASLD HCC, *P* = 0.934). The proportion of patients with an albumin–bilirubin score of 1 was higher in the MASLD group (*n* = 86, 48.9%) compared to non‐MASLD HCC (*n* = 193, 37.8%) (*P* = 0.010). Comparable proportions of people had a performance status of 0: 109 (62.0%) patients with MASLD HCC and 327 (64.0%, *P* = 0.872) patients with other causes of HCC.

**Table 3:** Stage of liver disease and performance status at the time of HCC diagnosis according to disease etiology

	Overall cohort	MASLD	Non‐MASLD	*P* value
Stage of liver disease at the time of HCC diagnosis				
Cirrhosis, *n* (%)	530 (77.1)	113 (64.2)	417 (81.6)	< 0.001
Ascites, *n* (%)	177 (25.8)	39 (22.2)	138 (27.0)	0.200
*Ascitic status unknown*	1 (0.1)	0 (0.0)	1 (0.2)	
Hepatic encephalopathy, *n* (%)	32 (4.7)	5 (2.9)	27 (5.3)	0.173
*Hepatic encephalopathy unknown*	16 (2.3)	2 (1.1)	14 (2.7)	
MELD‐Na score in people with cirrhosis				
MELD‐Na score, median (IQR)	9.8 (6.8)	9.5 (5.9)	9.8 (7.0)	0.815
MELD‐Na score ≤ 6, *n* (%)	76 (14.3)	16 (14.2)	60 (14.4)	0.919
MELD‐Na score < 9, *n* (%)	253 (47.7)	54 (47.8)	119 (47.7)	0.934
*MELD‐Na score unknown*	9 (1.7)	1 (0.6)	8 (1.9)	
ECOG performance status				
0, *n* (%)	436 (63.5)	109 (62.0)	327 (64.0)	0.872
1, *n* (%)	74 (10.8)	18 (10.2)	56 (11.0)	0.855
2, *n* (%)	86 (12.5)	23 (13.1)	63 (12.3)	0.722
3, *n* (%)	40 (5.8)	11 (6.3)	29 (5.7)	0.729
4, *n* (%)	15 (2.2)	3 (1.7)	12 (2.3)	0.639
*ECOG performance status unknown*	36 (5.2)	12 (6.8)	24 (4.7)	
ALBI grade				
Grade 1, *n* (%)	279 (40.6)	86 (48.9)	193 (37.8)	0.010
Grade 2, *n* (%)	317 (46.1)	74 (42.0)	243 (47.6)	0.206
Grade 3, *n* (%)	83 (12.1)	16 (9.1)	67 (13.1)	0.158
*ALBI grade unknown*	8 (1.2)	0 (0.0)	8 (1.6)	

ALBI grade, albumin–bilirubin grade; ECOG performance status, Eastern Cooperative Oncology Group performance status; HCC, hepatocellular carcinoma; MASLD, metabolic dysfunction‐associated steatotic liver disease; MELD, model for end‐stage liver disease.

### Patients with metabolic dysfunction‐associated steatotic liver disease hepatocellular carcinoma are less likely to receive treatment with curative intent

Overall, 14 (8.0%), 3 (1.7%), and 33 (18.8%) people with MASLD HCC underwent resection, liver transplant, or ablation as primary treatment, respectively, compared to 28 (5.5%), 24 (4.7%), and 146 (28.6.1%) people with HCC not related to MASLD (Table 4). Thus, the overall frequency of treatment delivered that had curative intent was 28.5% (*n* = 50) for MASLD HCC and 38.8% (*n* = 198) (*P* = 0.014) for non‐MASLD HCC. This trend was the same for planned primary treatment. Logistic regression identified that patients with MASLD HCC had 0.62 reduced odds (95% CI 0.43–0.91) of receiving curative treatment compared to non‐MASLD HCC (*P* = 0.014). However, on adjustment for the diameter of the largest lesion and the number of HCC nodules, statistically significant differences were not observed between MASLD and non‐MASLD patients (Table S6).

**Table 4:** Primary hepatocellular carcinoma treatment planned and delivered according to disease etiology

	Overall cohort	MASLD	Non‐MASLD	*P* value
Primary treatment planned				
Resection, *n* (%)	49 (7.1)	13 (7.4)	36 (7.0)	0.885
Transplant, *n* (%)	51 (7.4)	7 (4.0)	44 (8.6)	0.042
Ablation/PEI/IRE, *n* (%)	192 (27.9)	34 (19.3)	158 (30.9)	0.003
Therapy with curative intent (all), *n* (%)	292 (42.5)	54 (30.7)	238 (46.6)	< 0.001
TACE/SIRT, *n* (%)	160 (23.3)	65 (36.9)	95 (18.6)	< 0.001
Sorafenib/other chemotherapy/immunotherapy, *n* (%)	73 (10.6)	29 (16.5)	44 (8.6)	0.004
Best supportive care, *n* (%)	153 (22.3)	26 (14.8)	127 (24.9)	0.005
*Primary treatment planned unknown*	9 (1.3)	2 (1.1)	7 (1.4)	
Primary treatment delivered				
Resection, *n* (%)	42 (6.1)	14 (8.0)	28 (5.5)	0.233
Transplant, *n* (%)	27 (3.9)	3 (1.7)	24 (4.7)	0.079
Ablation/PEI/IRE, *n* (%)	179 (26.1)	33 (18.8)	146 (28.6)	0.011
Therapy with curative intent (all), *n* (%)	248 (36.2)	50 (28.5)	198 (38.8)	0.014
TACE/SIRT, *n* (%)	147 (21.4)	50 (28.4)	97 (19.0)	0.008
Sorafenib/other chemotherapy/immunotherapy, *n* (%)	52 (7.6)	16 (9.1)	36 (7.0)	0.370
Best supportive care, *n* (%)	226 (32.9)	56 (31.8)	170 (33.3)	0.742
*Primary treatment delivered unknown*	14 (2.0)	4 (2.3)	10 (2.0)	

IRE, irreversible electroporation; MASLD, metabolic dysfunction‐associated steatotic liver disease; PEI, percutaneous ethanol injection; SIRT, selective internal radiation therapy; TACE, transarterial chemoembolization.

### Patients with metabolic dysfunction‐associated steatotic liver disease hepatocellular carcinoma and non‐metabolic dysfunction‐associated steatotic liver disease hepatocellular carcinoma have comparable 1‐ and 5‐year survival

At 1 year, 100 people (56.8%) with MASLD and 287 (56.2%) people with non‐MASLD HCC were alive. The number of people surviving to 5 years was *n* = 22 (12.5%) for people with MASLD HCC and *n* = 65 (12.7%) for people with non‐MASLD HCC. Cox proportional hazard modeling identified that a diagnosis of MASLD did not impact overall survival (hazard ratio 1.03, 95% CI 0.85–1.25, *P* = 0.748) (Table S7), as also demonstrated in Kaplan–Meier survival curves (Fig. S1).

## Discussion

In our cohort of 687 patients with HCC, people with MASLD HCC were less likely to have been in cancer surveillance, compared to those with other chronic liver diseases, as a result of undiagnosed cirrhosis and tumor development without pre‐existing cirrhosis. MASLD HCC patients presented with larger tumors and were less frequently eligible for curative intent treatment, despite having better baseline liver function and similar performance status. Nonetheless, overall survival for MASLD‐related and non‐MASLD‐related HCC was similar.

Meta‐analysis data support our finding that patients with MASLD HCC are less likely to have their cancer detected in a surveillance program compared to non‐MASLD HCC.^11^ In 632 patients with HCC managed in a UK tertiary referral center, 22.8% of patients with MASLD HCC had disease detected by surveillance, *versus* 32.0% and 46.2% for patients with ALD and HCV‐related HCC.^15^ A follow‐up analysis of 275 patients with HCV‐related HCC and 212 with MASLD‐related HCC from two UK transplant centers identified that patients with MASLD HCC were older, less likely to have cirrhosis, and presented with larger tumors.^16^ Patients with MASLD‐related HCC were less likely to be candidates for potentially curative treatment, yet overall survival remained comparable.^16^ Similarly, in the meta‐analysis by Tan *et al*.,^11^ overall survival for patients with HCC was similar between liver disease etiologies, despite patients with MASLD being older and having larger tumors.^15^ Our study corroborates these findings in a largely prospective cohort and is the first to report on the impact of undetected liver disease in the community on a lack of HCC surveillance in patients with MASLD. Regression analysis identifies that enrollment in surveillance increases the likelihood of detecting smaller lesions, which impacts eligibility for curative treatment methods. While overall survival is comparable (likely a result of the slower progression of MASLD towards liver decompensation compared to other chronic liver diseases), the impact of lower levels of surveillance on HCC survival remains unknown.

We identify that a barrier to patients with MASLD entering HCC surveillance is that nearly two‐thirds had undiagnosed chronic liver disease at the time of HCC presentation. European^17^, ^18^ and American guidelines advise performing a Fibrosis‐4 score in people with metabolic risk factors. Currently, widespread targeted screening for MASLD is not recommended in the UK for all patients with metabolic disease, with data lacking on cost‐effectiveness.^19^ Locally, only 1.5% of 26 090 people with diabetes had undergone fibrosis testing (29.7% of those tested had significant liver fibrosis or above).^20^ We highlight that the absence of a coherent approach towards targeted screening is a major reason why patients with MASLD are not enrolled in HCC surveillance programs. The National Institute for Health and Clinical Excellence has recently published guidelines on the use of fibroscan outside of secondary care to facilitate the earlier diagnosis of chronic liver disease^21^; however, significant investment is needed to implement widespread fibroscan use in this setting.

We also identify that a higher percentage of patients with MASLD develop HCC without pre‐existing cirrhosis; thus, they are not eligible for cancer surveillance.^12^ This may be driven by oncogenic factors including genetic susceptibility,^22^ obesity,^23^ and type 2 diabetes,^24^, ^25^ as well as oxidative, inflammatory, and apoptotic changes associated with hepatic steatosis and insulin resistance.^26^ It is not practical to include all MASLD patients in HCC surveillance, and there is a need to risk stratify which patients with MASLD are at the greatest risk of HCC. Data from a large observational study of people participating in a health screening program found that serum liver enzyme levels can help stratify HCC risk, cirrhosis,^27^ cardiovascular comorbidities, and overall and cardiometabolic survival.^28^ The American Gastroenterology Association proposes that patients with MASLD with evidence of advanced liver fibrosis or cirrhosis, determined by combining at least two non‐invasive tests, undergo HCC screening. However, there are no data to support the suggested test cut‐offs.^29^ There is thus an unmet need to develop novel HCC risk prediction tools for people with MASLD. Most HCC risk models (PAGE‐B,^30^ Veterans Health Affairs model,^31^ and aMAP^32^) were developed in cohorts in which viral hepatitis was the predominant etiology. The natural history of viral and non‐viral liver diseases is different, and the estimates of HCC risk in a score developed for a hepatitis B population are unlikely to be applicable for an MASLD population. Consortia, including DeLIVER, focused on the discovery of novel biomarkers, may provide new knowledge. Any risk prediction tool should be tested in a randomized control trial with cost‐effective analysis, although we acknowledge the substantial sample size and time needed to demonstrate survival benefits.

This study benefits from a well‐phenotyped cohort collated in a region with one of the highest rates of HCC and MASLD mortality in the UK. Over half of the data is prospective, and the dataset provides novel information on the barriers to HCC surveillance and a comprehensive description of liver disease and tumor stages. While some data were retrospective, there was no inherent selection bias as cases were identified through hepatopancreaticobiliary cancer multidisciplinary meeting records. While we did not identify a statistically significant difference in survival, we could not obtain the cause of death via electronic records and therefore could not analyze differences in HCC‐related deaths or other competing risks, including death from liver decompensation or cardiovascular disease (particularly relevant to people with MASLD). Given that patients with MASLD‐related HCC were diagnosed later, there is also the possibility of lead‐time bias. Finally, we could not reliably collect data on regular attendance for HCC surveillance scans, that is, physician referral and patient attendance, and this likely represents an additional barrier to early detection.

In conclusion, patients with MASLD are less likely to have their HCC detected within a surveillance program because of higher rates of undetected liver disease and pre‐cirrhotic HCC. Consequently, patients with MASLD‐related HCC are less likely to receive treatment with curative intent. Current surveillance recommendations are failing groups of patients at high risk of HCC, and better predictive models are needed to individualize entry into surveillance according to HCC risk. Future research should also explore the cost‐effectiveness of targeted fibrosis screening in people with metabolic disease to identify those at high risk of HCC that would benefit from HCC surveillance.

## Supporting information


**Table S1.** Frequency of people presenting with an HCC previously enrolled in an HCC surveillance program according to the aetiology of chronic liver disease and reasons for lack of HCC surveillance.
**Table S2.** Indication for magnetic resonance or computerized tomography imaging which led to the diagnosis of HCC.
**Table S3.** Linear regression analysis for the impact of MASLD aetiology on the log of the largest tumor diameter (n = 672).
**Table S4.** Logistic regression analysis for the impact of MASLD aetiology on presenting with HCC and a largest tumour diameter > 5 cm (n = 672).
**Table S5.** Negative binomial regression for the impact of MASLD aetiology on the number of HCC tumours (n = 672).
**Table S6.** Logistic regression analysis for the impact of MASLD aetiology on receiving a primary treatment for HCC that has curative intent (n = 668).
**Table S7.** Cox proportional hazards model displaying the impact of MASLD aetiology on overall survival for patients with HCC (n = 672).
**Figure S1.** Kaplan–Meir curves for the overall survival between people with MASLD‐related and non‐MASLD related HCC.

## Data Availability

Data are available on request to the research team.
