# Supplementary material for: Lower hepatocellular carcinoma surveillance in metabolic dysfunction‐associated steatotic liver disease: Impact on treatment eligibility
Source: J Gastroenterol Hepatol. 2024 Aug 27;39(12):2817–25. doi: 10.1111/jgh.16727 (PMC11660197; doi:10.1111/jgh.16727)
Supplement: Supplementary file 1 — Table S1. Frequency of people presenting with an HCC previously enrolled in an HCC surveillance program according to the aetiology of chronic liver disease and reasons for lack of HCC surveillance. Table S2. Indication for magnetic resonance or computerized tomography imaging which led to the diagnosis of HCC. Table S3. Linear regression analysis for the impact of MASLD aetiology on the log of the largest tumor diameter (n = 672). Table S4. Logistic regression analysis for the impact of MASLD aetiology on presenting with HCC and a largest tumour diameter > 5 cm (n = 672). Table S5. Negative binomial regression for the impact of MASLD aetiology on the number of HCC tumours (n = 672). Table S6. Logistic regression analysis for the impact of MASLD aetiology on receiving a primary treatment for HCC that has curative intent (n = 668). Table S7. Cox proportional hazards model displaying the impact of MASLD aetiology on overall survival for patients with HCC (n = 672). Figure S1. Kaplan–Meir curves for the overall survival between people with MASLD‐related and non‐MASLD related HCC. [file JGH-39-2817-s001.docx]

**Supplementary Table 1.** Frequency of people presenting with an HCC previously enrolled in an HCC surveillance program according to the aetiology of chronic liver disease and reasons for lack of HCC surveillance

|  |  | **Overall cohort** | **MASLD** | **Non-MASLD** | **P value** |
| --- | --- | --- | --- | --- | --- |
|  | **Patients enrolled in a surveillance programme prior to HCC diagnosis, n (%)** | **253 (36.8)** | **38 (21.6)** | **215 (42.1)** | **<0.001** |
| **Patient not enrolled in a surveillance programme prior to HCC diagnosis** | *Patients known to secondary care liver services prior to diagnosis of HCC, n (%)* | 63 (14.5) | 19 (13.8) | 44 (14.9) | 0.752 |
|  | Cirrhotic, n (%) | 55 (87.3) | 14 (73.7) | 41 (93.2) | 0.033 |
|  | Non-cirrhotic, n (%) | 8 (12.7) | 5 (26.3) | 3 (6.8) |  |
|  | *Patients not known to secondary care liver services prior to diagnosis of HCC, n (%)* | 370 (85.3) | 119 (86.2) | 251 (84.8) | 0.752 |
|  | Cirrhotic, n (%) | 228 (61.6) | 62 (52.1) | 166 (66.1) | 0.010 |
|  | Non-cirrhotic, n (%) | 142 (38.4) | 57 (47.9) | 85 (33.9) |  |
|  | Secondary care status unknown, n (%) | 1 (0.2) | 0 (0) | 1 (0.3) |  |

MASLD, metabolic dysfunction associated steatotic liver disease; HCC, hepatocellular carcinoma

**Supplementary Table 2.** Indication for magnetic resonance or computerized tomography imaging which led to the diagnosis of HCC

|  | **Overall cohort** | **MASLD** | **Non-MASLD** | **P value** |
| --- | --- | --- | --- | --- |
| Surveillance scan, n (%) | 186 (27.5) | 26 (14.8) | 160 (32.0) | <0.001 |
| Abnormal liver enzymes, n (%) | 164 (24.3) | 48 (27.3) | 116 (23.2) | 0.278 |
| Rising AFP, n (%) | 49 (7.2) | 6 (3.4) | 43 (8.6) | 0.022 |
| Incidental finding on other imaging suggestive of a liver lesion, n (%) | 129 (19.1) | 47 (26.7) | 82 (16.4) | 0.003 |
| Abdominal pain, n (%) | 45 (6.7) | 17 (9.7) | 28 (5.6) | 0.063 |
| Weight loss, n (%) | 28 (4.1) | 11 (6.3) | 17 (3.4) | 0.103 |
| Clinical ascites, n (%) * | 19 (2.8) | 1 (0.6) | 18 (3.6) | 0.034 |
| Hepatomegaly, n (%) * | 7 (1.0) | 2 (1.1) | 5 (1.0) | 1.00 |
| Anaemia, n (%) * | 6 (0.9) | 3 (1.7) | 3 (0.6 | 0.185 |
| Variceal bleed, n (%) * | 11 (1.6) | 5 (2.8) | 6 (1.2) | 0.165 |
| Other, n (%) | 32 (4.7) | 10 (5.7 | 22 (4.4) | 0.491 |

*2-sided fisher’s exact test

**Supplementary Table 3.** Linear regression analysis for the impact of MASLD aetiology on the log of the largest tumor diameter (n=672)

| **Variable** | **Coefficient (β)** | **Exponentiated coefficient** | **P-value** | **95% Confidence Interval** |
| --- | --- | --- | --- | --- |
| **Unadjusted Model** | | | | |
| Aetiology (MASLD) | 0.207 | 1.230 | 0.002 | 0.076, 0.338 |
| **Model 1** | | | | |
| Aetiology (MASLD) | 0.164 | 1.178 | 0.018 | 0.028, 0.299 |
| Age | 0.006 | 1.006 | 0.020 | 0.001, 0.012 |
| Gender (Male) | 0.140 | 1.150 | 0.041 | 0.006, 0.275 |
| **Model 2** | | | | |
| Aetiology (MASLD) | 0.206 | 1.229 | 0.005 | 0.062, 0.35 |
| Age | 0.007 | 1.007 | 0.013 | 0.001, 0.012 |
| Gender (Male) | 0.153 | 1.165 | 0.027 | 0.017, 0.288 |
| Type 2 Diabetes | -0.110 | 0.896 | 0.086 | -0.236, 0.016 |
| **Model 3** | | | | |
| Aetiology (MASLD) | 0.116 | 1.123 | 0.104 | -0.024, 0.255 |
| Age | 0.003 | 1.003 | 0.191 | -0.002, 0.009 |
| Gender (Male) | 0.098 | 1.103 | 0.141 | -0.033, 0.228 |
| Type 2 Diabetes | -0.062 | 0.940 | 0.313 | -0.183, 0.059 |
| Surveillance status | -0.464 | 0.629 | <0.001 | -0.582, -0.346 |

Model 1: Adjusted for age, sex

Model 2: Adjusted for age, sex, diabetes status

Model 3: Adjusted for age, sex, diabetes status, HCC surveillance status

**Supplementary Table 4.** Logistic regression analysis for the impact of MASLD aetiology on presenting with HCC and a largest tumour diameter > 5cm (n=672)

| **Variable** | **Odds Ratio (OR)** | **P-value** | **95% Confidence Interval** |
| --- | --- | --- | --- |
| **Unadjusted Model** | | | |
| Aetiology (MASLD) | 1.882 | <0.001 | 1.306, 2.711 |
| **Model 1** | | | |
| Aetiology (MASLD) | 1.595 | 0.016 | 1.089, 2.335 |
| Age | 1.026 | <0.003 | 1.009, 1.043 |
| Gender (Male) | 1.091 | 0.667 | 0.733, 1.625 |
| **Model 2** | | | |
| Aetiology (MASLD) | 1.835 | <0.004 | 1.214, 2.774 |
| Age | 1.027 | <0.002 | 1.010, 1.044 |
| Gender (Male) | 1.133 | 0.542 | 0.758, 1.693 |
| Type 2 Diabetes | 0.704 | 0.70 | 0.482, 1.029 |
| **Model 3** | | | |
| Aetiology (MASLD) | 1.522 | 0.054 | 0.993, 2.332 |
| Age | 1.018 | 0.036 | 1.001, 1.035 |
| Gender (Male) | 0.994 | 0.976 | 0.655, 1.508 |
| Type 2 Diabetes | 0.776 | 0.206 | 0.524, 1.150 |
| Surveillance status (Enrolled) | 0.275 | <0.001 | 0.180, 0.420 |

Model 1: Adjusted for age, sex

Model 2: Adjusted for age, sex, diabetes status

Model 3: Adjusted for age, sex, diabetes status, HCC surveillance status

**Supplementary Table 5.** Negative binomial regression for the impact of MASLD aetiology on the number of HCC tumours (n=672)

| **Variable** | **Incidence Rate Ratio** | **P-value** | **95% Confidence Interval** |
| --- | --- | --- | --- |
| **Unadjusted Model** | | | |
| Aetiology (MASLD) | 1.024 | 0.716 | 0.900, 1.166 |
| **Model 1** | | | |
| Aetiology (MASLD) | 1.050 | 0.481 | 0.918, 1.200 |
| Age | 0.996 | 0.192 | 0.991, 1.002 |
| Gender (Male) | 1.086 | 0.238 | 0.947, 1.245 |
| **Model 2** | | | |
| Aetiology (MASLD) | 1.059 | 0.433 | 0.918, 1.222 |
| Age | 0.997 | 0.207 | 0.991, 1.002 |
| Gender (Male) | 1.089 | 0.226 | 0.949, 1.249 |
| Type 2 Diabetes | 0.977 | 0.722 | 0.861, 1.109 |
| **Model** **3** | | | |
| Aetiology (MASLD) | 1.021 | 0.782 | 0.883, 1.179 |
| Age | 0.995 | 0.088 | 0.990, 1.001 |
| Gender (Male) | 1.063 | 0.386 | 0.926, 1.219 |
| Type 2 Diabetes | 0.997 | 0.963 | 0.879, 1.131 |
| Surveillance status | 0.825 | 0.003 | 0.728, 0.935 |

Model 1: Adjusted for age, sex

Model 2: Adjusted for age, sex, diabetes status

Model 3: Adjusted for age, sex, diabetes status, HCC surveillance status

**Supplementary Table 6**. Logistic regression analysis for the impact of MASLD aetiology on receiving a primary treatment for HCC that has curative intent (n=668)

| **Variable** | **Odds Ratio (OR)** | **p-value** | **95% Confidence Interval** |
| --- | --- | --- | --- |
| **Unadjusted Model** | | | |
| Aetiology (MASLD) | 0.624 | 0.014 | 0.429, 0.908 |
| **Adjusted Model** | | | |
| Aetiology (MASLD) | 0.722 | 0.124 | 0.476, 1.094 |
| Diameter of largest lesion | 0.975 | <0.001 | 0.968, 0.982 |
| No HCC nodules | 0.625 | <0.001 | 0.540, 0.725 |

**Supplementary Table 7.** Cox proportional hazards model displaying the impact of MASLD aetiology on overall survival for patients with HCC (n=672)

| **Variable** | **Hazard Ratio (HR)** | **P-value** | **95% Confidence Interval** |
| --- | --- | --- | --- |
| **Unadjusted Model** | | | |
| Aetiology (MASLD) | 1.032 | 0.748 | 0.853, 1.248 |
| **Model 1** | | | |
| Aetiology (MASLD) | 1.013 | 0.894 | 0.834, 1.231 |
| Age | 1.003 | 0.431 | 0.995, 1.011 |
| Gender (Male) | 0.933 | 0.501 | 0.763, 1.141 |
| **Model 2** | | | |
| Aetiology (MASLD) | 1.026 | 0.805 | 0.836, 1.260 |
| Age | 1.003 | 0.410 | 0.995, 1.012 |
| Gender (Male) | 0.937 | 0.529 | 0.766, 1.147 |
| Type 2 Diabetes | 0.965 | 0.703 | 0.804, 1.158 |
| **Model 3** | | | |
| Aetiology (MASLD) | 0.909 | 0.372 | 0.737, 1.121 |
| Age | 0.999 | 0.833 | 0.991, 1.007 |
| Gender (Male) | 0.898 | 0.297 | 0.733, 1.100 |
| Type 2 Diabetes | 1.025 | 0.793 | 0.853, 1.232 |
| Surveillance status | 0.639 | <0.01 | 0.529, 0.772 |

**Supplementary Figure 1.** Kaplan Meir curves for the overall survival between people with MASLD-related and non-MASLD related HCC
